# Hybrid-Enhanced Siamese Similarity Models in Ligand-Based Virtual Screen

**DOI:** 10.3390/biom12111719

**Published:** 2022-11-20

**Authors:** Mohammed Khaldoon Altalib, Naomie Salim

**Affiliations:** 1 School of Computing, Universiti Teknologi Malaysia, Johor Bahru 81310, Malaysia; 2 Computer Science Department, Education for Pure Science Collage, University of Mosul, Mosul 41002, Iraq; 3 UTM Big Data Centre, Ibnu Sina Institute for Scientific and Industrial Research, Universiti Teknologi Malaysia, Johor Bahru 81310, Malaysia

**Keywords:** drug discovery, ligand-based virtual screen, similarity model, Siamese architecture, hybrid model

## Abstract

Information technology has become an integral aspect of the drug development process. The virtual screening process (VS) is a computational technique for screening chemical compounds in a reasonable amount of time and cost. The similarity search is one of the primary tasks in VS that estimates a molecule’s similarity. It is predicated on the idea that molecules with similar structures may also have similar activities. Many techniques for comparing the biological similarity between a target compound and each compound in the database have been established. Although the approaches have a strong performance, particularly when dealing with molecules with homogenous active structural, they are not enough good when dealing with structurally heterogeneous compounds. The previous works examined many deep learning methods in the enhanced Siamese similarity model and demonstrated that the Enhanced Siamese Multi-Layer Perceptron similarity model (SMLP) and the Siamese Convolutional Neural Network-one dimension similarity model (SCNN1D) have good outcomes when dealing with structurally heterogeneous molecules. To further improve the retrieval effectiveness of the similarity model, we incorporate the best two models in one hybrid model. The reason is that each method gives good results in some classes, so combining them in one hybrid model may improve the retrieval recall. Many designs of the hybrid models will be tested in this study. Several experiments on real-world data sets were conducted, and the findings demonstrated that the new approaches outperformed the previous method.

## 1. Introduction

Drug discovery often involves multiple stages, beginning with the identification of a biological target, followed by the parallel screening of thousands of compounds and, finally, the production of the new drug. This technique is time-consuming, costly, and plagued with numerous difficulties. Virtual screening (VS) is a drug discovery computational technique that searches libraries of molecules for structures that are most able to belong to a drug target at a reasonable cost and time. Virtual screening is classified into two types: structure-based approaches, such as ligand-protein docking, and ligand-based approaches, such as similarity searching, machine learning, and pharmacophore mapping [1,2,3,4,5]. Similarity searching is the most effective and one of the maximal broadly used equipment for ligand-based virtual screening because it requires only a bioactive molecule, or reference structure, as the point to begin a database search. The fundamental concept underlying similarity searching states that structurally compatible molecules will show off similar physicochemical and organic properties. A similarity search compares target structure characteristics with each structure’s attributes in the database. The degree of resemblance of these two sets of features is used to measure the degree of closeness. Then, the database structures are usually ranked in decreasing order of similarity to the target. When discussing LBVS, various elements must be considered, including molecular representation and similarity coefficients, among others [6,7,8,9,10,11]. In cheminformatics, fingerprints are a crucial and widely used concept. Their primary goal is to create numerical representations of molecules’ structure or specific properties, allowing the comparison of two molecules to be quantified [1]. Molecular characteristics are spread from physicochemical attributes to structural features and are stored in various methods, referred to as molecular descriptors. The molecular descriptor aims to capture the most important features of the molecule. To compare the fingerprints of two chemicals, various similarity metrics can be used, such as Euclidean distance, Manhattan distance, and Dice coefficient, but the Tanimoto coefficient is the most preferred [5].

In recent years, data fusion has gained acceptance as one of the methods for improving the performance of existing systems for ligand-based virtual screening. Data fusion is the process of integrating numerous data sources into a single source using fusion techniques, assuming that the outcomes of the fused source will be more valuable than the individual input sources. For example, when many similarity coefficients were combined, it became more active than when individual coefficients were employed [1,4,12,13]. Dasarathy presented one of the most well-known data fusion classification systems, which is made up of the following five categories: (1) Data in-Data out, in which the raw data is inputted and outputted, the outcomes are often more accurate or dependable; (2) Data in-Feature out, in which the data fusion method uses the raw data from various participants at this level to extract the features or characteristics that describe an object or class; (3) Feature in-Feature out, in which the input and output of the data fusion procedure here are the features to enhance, hone, or create new features; (4) Feature in-Decision out, this level produces a set of decisions based on a collection of features that are obtained as input; (5) Decision In-Decision Out: is also called decision fusion, to fuse the input decisions and produce superior or novel judgments [14].

Several approaches were concerned with enhancing and increasing the retrieval effectiveness of the methods of similarity searching and the ways to calculate them. Several efforts were taken to improve and increase the retrieval efficacy of similarity searching methods, concluding that the Tanimoto coefficient is the industry-standard and outperforms others [15,16,17,18]. Some studies sought to include approaches from text document retrieval and apply them to molecular searches, such as Abdo et al., who used a Bayesian network that was originally used in the text field in document retrieval, and modified it as the retrieval model in the cheminformatic area [6]. Furthermore, Al Dabagh et al. applied quantum mechanics physics concepts to improve the molecular similarity searching and molecular ranking of chemical compounds in LBVS [9]. Some researchers, such as Ahmed et al., are working on weighting approaches to increase the retrieval effectiveness of a Bayesian inference network, allowing more weights to be added to relevant fragments while deleting the unnecessary ones [19,20,21]. Some studies looked into data fusion and proposed that similarity measurements be merged by combining the screening results obtained by employing multiple similarity measures. Nasser et al. fused several descriptors by selecting the best features from each descriptor and then merging them in the new descriptor [3,4,22]. Although the above methods outperform their predecessors, particularly when dealing with molecules with homogeneous active structural elements such as molecules’ classes in MDDR-DS2 dataset as will shown in Section 3.1, the performances are not good or satisfactory when dealing with molecules with a structurally heterogeneous nature such as molecules’ classes in MDDR-DS3 dataset as will shown in Section 3.1.

On the other hand, The Siamese network has been used for more complicated data samples, especially with heterogeneous data samples, and it is possible to employ deep learning methods with Siamese architecture, which deals efficiently with the vast volume of information stored in databases [23,24]. Altalib et al. employed many deep learning methods in Siamese architecture after enhancing with two similarity measures and one fusion layer to improve the retrieval effectiveness of molecules that have a structurally heterogeneous nature. The first study employed four methods of deep learning in Siamese architecture, which are: Gated Recurrent Unit (GRU), Long Short-Term Memory (LSTM), Convolutional Neural Network-two dimensions (CNN-2D), and Convolutional Neural Network-one dimension (CNN-1D). The second study employed Multilayer Perceptron (MLP) in Siamese architecture [25,26]. This study continues to improve the effectiveness of similarity retrieval for molecules that have structurally heterogeneous by incorporating the two best previous models into one hybrid model. The reason is that each method gives good results in some classes, so combining them in one hybrid model may improve the retrieval recall. The following are the main contributions of this study:

- The Siamese architecture for selected methods will be enhanced with three similarity measures to better improve the similarity measurements between molecules.
- Incorporate many designs of a hybrid model from the selected two models. As mentioned before, each method gives good results in some classes, so combining them in one hybrid model may improve the retrieval recall.
- Compared to previous approaches, the proposed strategy yielded promising results in terms of overall performance, particularly when dealing with heterogeneous classes of molecules.

## 2. Methods

### 2.1. Siamese Architecture

**Figure 1:**
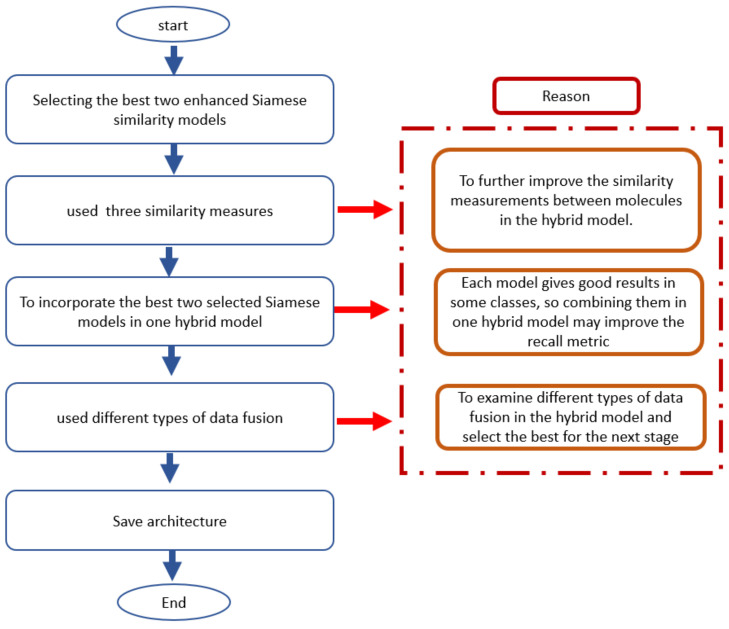
The steps for incorporating two enhanced Siamese similarity models into one hybrid model.

Two identical artificial neural networks made up a Siamese neural network, each capable of handling the input data, which must be coupled to a last layer via a distance layer to foresee whether the two vectors belong to the same class. Because all the weights and biases in the Siamese architecture are related, they are referred to as twins. Both networks are symmetric as a result of this. Through training, the two neural networks also utilize feedforward perceptron and error backpropagation. As a result, it has been used on more complicated data samples, such as heterogeneous data samples with different dimensions and type attributes [23,24]. This work is considered extensions of our previous work [25,26]. Figure 1 shows steps for incorporating two enhanced Siamese similarity models into one hybrid model.

The main goal of this work is to improve the retrieval efficiency of molecular similarity searching, especially with structurally heterogeneous molecules, by incorporating two enhanced Siamese deep learning similarity models in one hybrid model. Thus, the steps for incorporating two enhanced Siamese similarity models in one hybrid model will be explained as follows:

1. We select the best two enhanced Siamese deep learning models from the previous studies according to the Kendall W significance test for ranking the methods. The two best methods in MDDR-DS3 (structurally heterogeneous) are the Siamese multi-layer perceptron similarity model (SMLP) and Siamese convolutional neural network -one dimension (SCNN1D) similarity model.
2. The Siamese architecture in the selected models will be enhanced with three similarity measures. The reason for this is to further improve the measurements of similarity between molecules in the hybrid model.
3. Incorporate the selected best two models in a hybrid model. Since each model gives good results in some classes, combining them in one hybrid model may improve the retrieval recall.
4. Testing many designs of hybrid models by using different types of data fusion to select the best hybrid model that will give good results of the recall metric when using with structurally heterogeneous molecules dataset. We select the best two enhanced Siamese deep learning models from the previous phase according to the Kendall W significance test for ranking the methods.

### 2.2. Hybrid Siamese Similarity Model Using Decision Fusion

The first design of the hybrid similarity model combines the two selected models from the previous studies. The first model is the SMLP similarity model, and the second is the SCNN1D similarity model. The reason is that each method gives good results in some classes, so combining them in one hybrid model may improve the retrieval recall. The architecture of the SMLP consists of two twin neural networks, each of which has only one layer with 1024 neurons. The weights have also been linked in this architecture so that Network1 = Network2. The first network reads the fingerprint from the query, and the second reads the fingerprint from the database. The output of each network is a features vector with a fixed length (here, 1024 features). The first similarity measure is the absolute difference between the two feature vectors, and the output of this measure is the vector of length 1024. The formula of this measure is [27]:

(1) $$S_{AB}=\left|f_{A}-f_{B}\right|.\;$$

where *S_AB_* is the similarity measure, *f_A_* is the feature victor of network-1, and *f_B_* is the feature vector of network-2. The second similarity measure is the exponential Manhattan distance [28]. The output of this measure is one value. The formula of the exponential Manhattan is:

(2) $$E_{AB}=exp\left(-\sum \left|f_{A}-f_{B}\right|\right).\;$$

where *E_AB_* is the exponential Manhattan distance, *f_A_* is the feature vector of network1, and *f_B_* is the feature vector of network2. The fusion layer has added the value of the second similarity measure with each value of the vector of the first similarity measure. The output of the fusion layer is passed to the following layers, which contain 1024, 512, 256, 128, and 64 neurons, respectively. Each neuron is connected with all neurons in the previous layer. The ReLU activation function has been used for all layers. The Siamese architecture ends with the output layer, which contains the active sigmoid function that gives the similarity score if 1 means complete similarity and if 0 means complete dissimilarity. Moreover, the RMSprop optimizer has been used, and the binary_crossentropy has been used as a loss function. The architecture of the SCNN1D similarity model consists of two twin neural networks, each of which has two layers of convolution neural network (1D-CNN). The layers are made up of 64 filters with a kernel size of 3; the activation function is a rectified linear unit (ReLU), and the maximum pooling size is 2. Then, there comes a flattened layer, followed by a dense layer with a sigmoid activation function. In this design, the weights are connected so that CNN1D-1 = CNN1D-2. The output of each network is a features vector with a fixed length (here, 512 features). The first similarity measure is the absolute difference between the two feature vectors, and the output of this measure is the vector of length 512. The second similarity measure is the exponential Manhattan distance. The output of this measure is one value. The fusion layer has added the value of the second similarity measure with each value of the vector of the first similarity measure. The output of the fusion layer is passed to the end layer, which contains the active sigmoid function that gives the similarity score if 1 means complete similarity and if 0 means complete dissimilarity. Moreover, the RMSprop optimizer has been used, and the binary_crossentropy has been used as a loss function.

**Figure 2:**
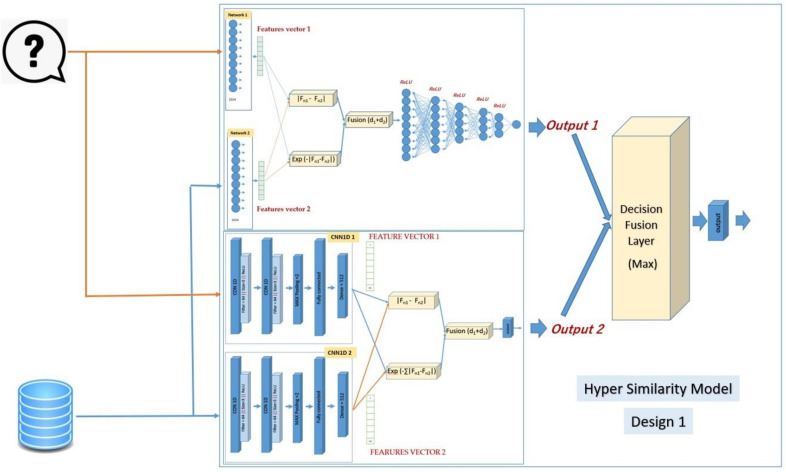
The design of the hybrid Siamese similarity model with two similarity measures using decision fusion.

The SMLP similarity model’s output (similarity score) will be fused with the output of the SCNN1D similarity model by using decision fusion (maximum). Figure 2 shows the details of the design of the hybrid Siamese similarity model with two similarity measures using the decision fusion.

### 2.3. Hybrid Siamese Similarity Model with Three Similarity Measures Using Decision Fusion

**Figure 3:**
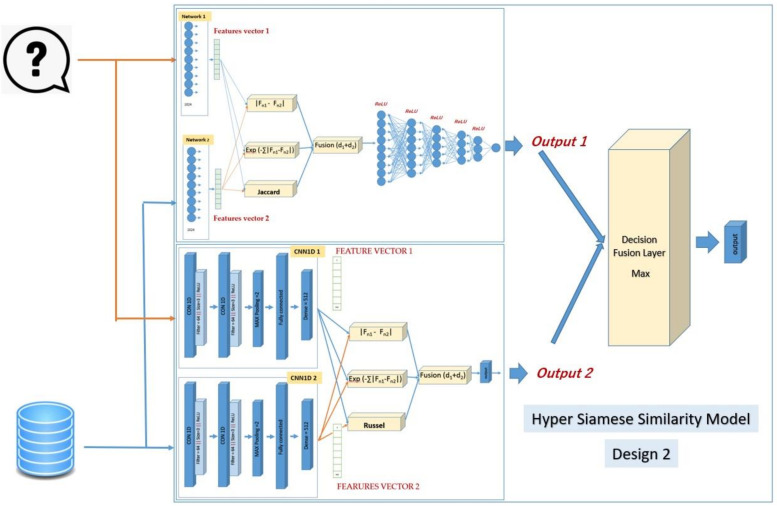
The design of the hybrid Siamese similarity model with three similarity measures using decision fusion.

The second design of the hybrid similarity model same as the first design of the hybrid similarity model except using three similarity measures instead of two similarity measures. The second design of the hybrid similarity model is the same as the first design of the hybrid similarity model, except using three similarity measures instead of two similarity measures. The first is the SMLP similarity model, and the second is the SCNN1D similarity model. The third similarity measure will be added for each of them. The reason for that is it further improves the measurements between molecules. The Jaccard similarity measure will be added to the SMLP similarity model as the third similarity measure. The Russel similarity measure will be added to the SCNN1D similarity model as the third similarity measure. The selection for these measures is based on experiments. The formula of the Jaccard similarity measure [29]:

(3) $$\delta_{AB}=\frac{\sum_{i=1}^{N}f_{iA}f_{iB}}{\sum_{i=1}^{N}{\left(f_{iA}\right)}^{2}+\sum_{i=1}^{N}{\left(f_{iB}\right)}^{2}-\sum_{i=1}^{N}f_{iA}f_{iB}}$$

where the features of the query’s molecular is *fi_A_*, the features of the dataset’s molecular is *fi_B_*, and *N* is the number of features in the vector. The formula of the Russel similarity measure [29] is:

(4) $$\delta_{AB}=\frac{\sum_{i=1}^{N}f_{iA}f_{iB}}{n}$$

where *fi_A_* is the features of the query’s molecular, *fi_B_* is the features of the dataset’s molecular, and *n* is the number of features. The SMLP similarity model’s output (similarity score) will be fused with the output of the SCNN1D similarity model by using the decision fusion (maximum). Figure 3 shows the details of the design of the hybrid Siamese similarity model with three similarity measures using the decision fusion.

### 2.4. Hybrid Siamese Similarity Model with Three Similarity Measures Using Feature Fusion Summation

The third design of the hybrid similarity model combines the two selected models: the first is the SMLP similarity model, and the second is the SCNN1D similarity model. The architecture here of the SMLP consists of two twin neural networks, each of which has only one layer with 1024 neurons. The weights have also been linked in this architecture so that Network1 = Network2. The first network reads the fingerprint from the query, and the second reads the fingerprint from the database. The output of each network is a features vector with a fixed length (here, 1024 features). The first similarity measure is the absolute difference between the two feature vectors, and the output of this measure is the vector of length 1024. The second similarity measure is the exponential Manhattan distance. The output of this measure is one value. The two formulas were covered in Section 2.2. The third similarity measure is the Jaccard measure. The formula was covered in Section 2.3. The output of this measure is one value. The feature fusion layer has added the value of the third similarity measure with the value of the second similarity measure. Then, the result has added with each value of the vector of the first similarity measure. The output of the fusion layer is passed to the following layers, which contain 1024 512 neurons, respectively. Each neuron is connected with all neurons in the previous layer. The ReLU activation function has been used for all layers. The output of this model is a features vector with a length of 512.

**Figure 4:**
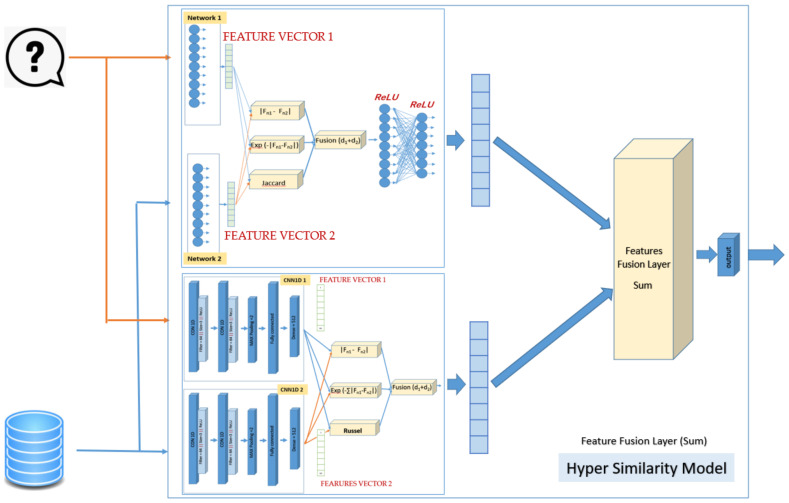
The design of the hybrid Siamese similarity model with three similarity measures using feature fusion summation.

The architecture of the SCNN1D similarity model consists of two twin neural networks, each of which has two layers of the convolution neural network (CNN1D). The layers are made up of 64 filters with a kernel size of 3; the activation function is a rectified linear unit (ReLU), and the maximum pooling size is 2. Then comes a flattened layer, followed by a dense layer with a sigmoid activation function. In this design, the weights are connected so that CNN1D-1 = CNN1D-2. The output of each network is a features vector with a fixed length (here, 512 features). The first similarity measure is the absolute difference between the two feature vectors, and the output of this measure is the vector of length 512. The second similarity measure is the exponential Manhattan distance. The output of this measure is one value. The third similarity measure is that the Russel similarity measure will be added to the SCNN1D similarity model as the third similarity measure. The feature fusion layer has added the value of the third similarity measure with the value of the second similarity measure. Then, the result has added with each value of the vector of the first similarity measure. The output of the fusion layer is the feature vector, which is considered as the output of second model. Figure 4 shows the details of the design of the hybrid Siamese similarity model with three similarity measures using feature fusion summation.

The SMLP similarity model’s output (feature vector 512 bit) will be fused with the output of the SCNN1D similarity model (feature vector 512 bit) by using feature fusion (sum). The result of this layer will be passed to the last layer of the hybrid model, which contains the active sigmoid function that gives the similarity score if 1 means complete similarity and if 0 means complete dissimilarity. Moreover, the RMSprop optimizer has been used, and the binary_crossentropy has been used as a loss function.

### 2.5. Hybrid Siamese Similarity Model with Three Similarity Measures Using Feature Fusion Maximum

**Figure 5:**
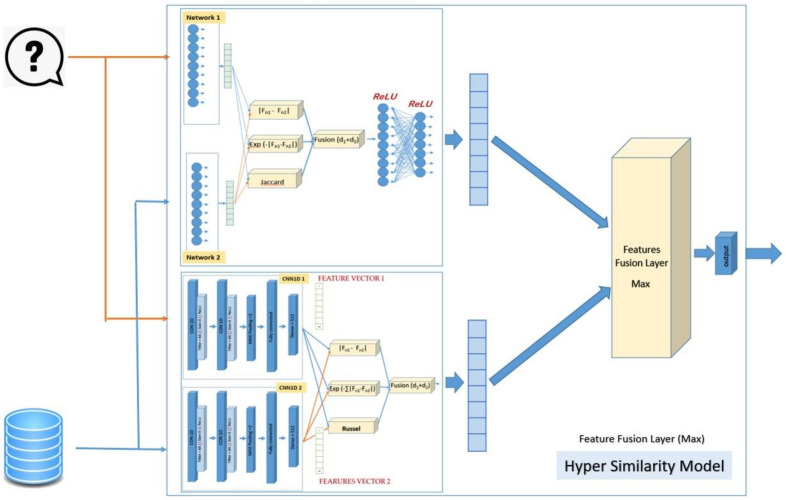
The design of the hybrid Siamese similarity model with three similarity measures using feature fusion maximum.

The fourth design of the hybrid similarity model is similar to the previous design (third design), except using maximum operation instead of sum operation in the feature fusion between the SMLP similarity model and the SCNN1D similarity model in the hybrid model. Figure 5 shows the details of the design of the hybrid Siamese similarity model with three similarity measures using the feature fusion maximum.

## 3. Experimental Design

### 3.1. Datasets

Here, we evaluate the search methods for similarity by using MDL Drug Data Report (MDDR) and the Maximum Unbiased Validation (MUV) data sets, which are the most common [30,31]. The MDDR datasets have been used by our research group and previous studies [3,4,6,9,10,19,20,21,22,25,26,32,33,34,35,36,37,38,39,40]. All molecules have been translated to ECFC-4 fingerprint by the Pipeline Pilot software, and our study community has used these databases. Ten reference structures were chosen randomly from each activity class. The MDDR contains three types of data sets, which are:

1. MDDR-DS1: This consists of 102,516 molecules divided into activity and inactivity groups. The activity molecules are split into 11 categories, with some having structurally homogeneous active elements and others having structurally heterogeneous active elements. Table 1 shows the activity classes of molecules in DS1.
2. MDDR-DS2: This contains 102,516 molecules divided into activity and inactivity groups. The activity molecules are split into 10 groups of homogeneous activity classes activity molecules. Table 2 shows the activity classes of molecules in DS2.
3. This contains 102,516 molecules divided into activity and inactivity groups; the activity molecules are split into 10 groups of heterogeneous activity classes activity molecules. Table 3 shows the activity classes of molecules in DS3.

Rohrer and Baumann documented the data gathering (MUV), as observed in Table 4. This data collection contains 17 interaction groups, with each class including up to 30 active and 15,000 inactive molecules. Our research team has utilized these data collections in prior papers.

### 3.2. Performance Evaluation Measures

**Figure 6:**
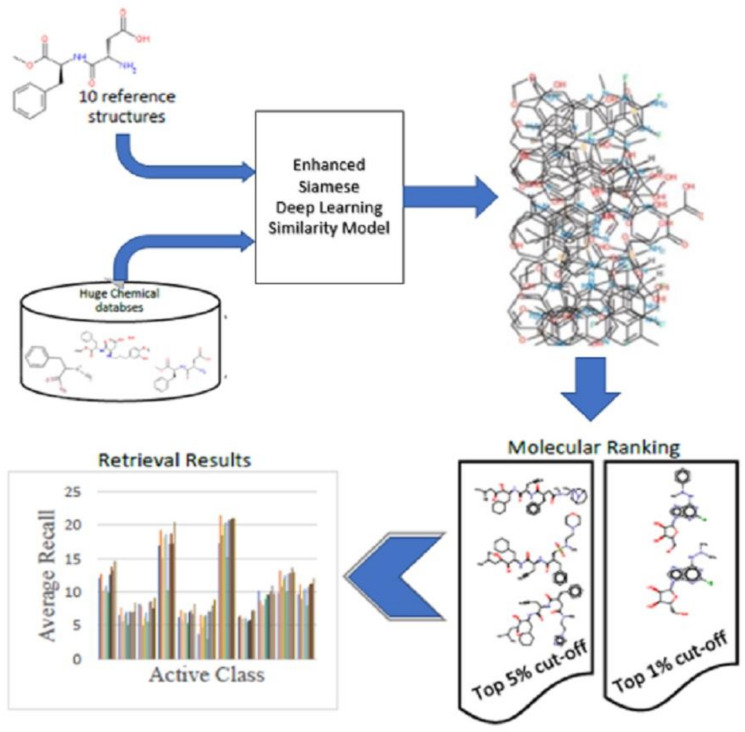
The general steps of the experimental design of this study.

The effectiveness of the proposed approaches is assessed as follows:

1. The recall metric, which is the part of active chemical compounds that can be identified inside the top 1 and 5% of the ranking test set, is the first method for assessing the retrieval model’s performance. This metric has already been utilized in research [3,4,6,9,10,19,20,21,22,25,26,32,33,34,35,36,37,38,39,40]. Figure 6 shows the general steps of the experimental design of this study.

**Figure 7:**
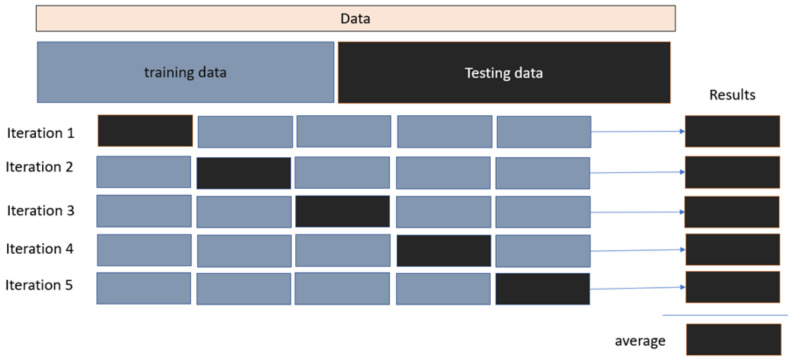
The cross validation for training and testing data.

Here, the whole dataset is separated into K equal-sized sets, one of which is designated as a test set and the rest sets as training sets. The test set is changed in each iteration, and the final result is determined as the average of the recall values from all iterations. As observed in Figure 7, this procedure is known as k-fold cross-validation. Each iteration tests 10 questions chosen at random from the activity class, and the mean value of these ten searches is determined.

2. Comparison Methods: The second strategy is to look at existing approaches that could be used to evaluate the outcomes of the proposed models and that use the same datasets. Among these approaches are the following:
  A. Tanimoto similarity coefficient (TAN): for many years, TAN has served as the LBVS search benchmark technique. Tanimoto-based similarity models use the Tanimoto coefficient in its continuous version for the ECFC-4 descriptor [15].
  B. Bayesian inference (BIN), the second method is BIN for the ECFC-4 descriptor [6].
  C. Quantum similarity search (SQB); the third method is SQB, which utilizes a quantum mechanics approach for the ECFC-4 descriptor [9].
  D. Stack of Deep Belief Networks (SDBN): The latest study is multi-descriptor-based on the Stack of deep belief networks method at the MDDR dataset (DS1, DS2, and DS3) for ECFC-4, ECFP-4, and EPFP-4 descriptors. The molecular features were reweighted using deep belief networks [4].
  E. Enhanced Siamese Convolutional Neural network—one dimension (SCNN-1D) [26] and Enhanced Siamese Multilayer perception (SMLP) [25], which are compared with them before and after being combined into one hybrid model.
3. The Kendall W is the third significant measure that may be used to evaluate the suggested procedures, often known as the significance test. This significance test has already been utilized in prior research [3,4,9,10,19,20,21,22,25,26,29,32,33,34,36,37,38,39,40,41,42]. This test can be construed as a measure of rater agreement. In the Kendall W test, each case represents a judge or rater, and each variable represents an object or person being rated. The number of rankings is computed for each variable. The Kendall W test range is between (0), indicating no agreement, and (1) indicating full agreement. For example, the rank *rij* by judge number *j*, which represents an activity class, where there are *n* objects and *m* judges in total, is given to object *i* as the similarity search method. It is then possible to calculate the total rank given to object *i* as [43]:

(5) $${ℜ}_{i}=\sum_{j=1}^{m}r_{ij}$$

Whereas the complete ranks’ mean meaning is:

(6) $$\bar{ℜ}=\frac{1}{2}m\left(n+1\right)$$

Squared deviation sum *δ* is defined as:

(7) $$\delta =\sum_{i=1}^{n}{\left({ℜ}_{i}-ℜ\right)}^{2}$$

Then, the Kendall *W* test is defined as:

(8) $$W=\frac{12\delta }{m^{2}\left(n^{3}-n\right)}.\;$$

This test demonstrates whether a group of judges can make equivalent decisions about the rating of a set of items or not. The definitions used in this analysis suggest that judges were considered to be the behavior groups of each of the data sets, whereas the recall rates of the different search models were considered to be the items. The outcomes of the Kendall coefficient that are related to significance levels are a significant part of this experiment. This implies verifying whether the value of the coefficient may have happened by chance or not. If the value was important (for which both 0.01 and 0.05 cut-off values were used), it was then possible to assign the item an overall ranking.

4. For a more evident comparison between the recall values of the proposed methods and the recall values of the previous methods, the improvement percentage for each proposed method will be calculated. The improvement percentage formula is [44].

(9) $${\mathrm{Improvement}}_{method1}=\frac{{\;\mathrm{Recall}\;}_{\mathrm{method}1\;}–{\;\mathrm{Recall}\;}_{\mathrm{method}2\;}}{{\;\mathrm{Recall}\;}_{\mathrm{method}1\;}}\times 100\%$$

where the ${\;\mathrm{Recall}\;}_{\mathrm{method}1\;}$ represented the recall value of the first method, and ${\;\mathrm{Recall}\;}_{\mathrm{method}2\;}\;$ represented the recall value of second method.

## 4. Results and Discussion

**Figure 8:**
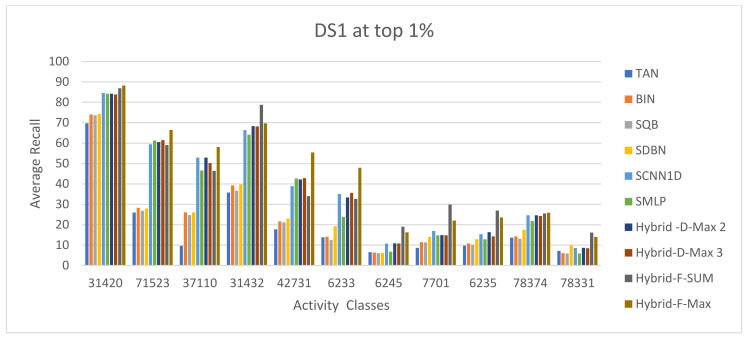
The comparison among methods for the average recall percentage at the top 1% in MDDR-DS1 (homogeneous and heterogeneous).

**Figure 9:**
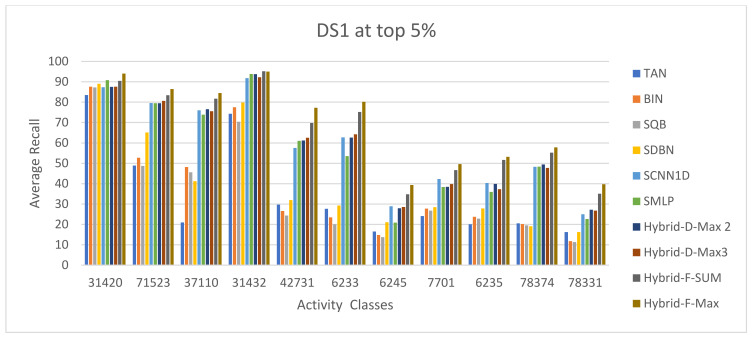
The comparison among methods for the average recall percentage at the top 5% in MDDR-DS1 (homogeneous and heterogeneous).

**Figure 10:**
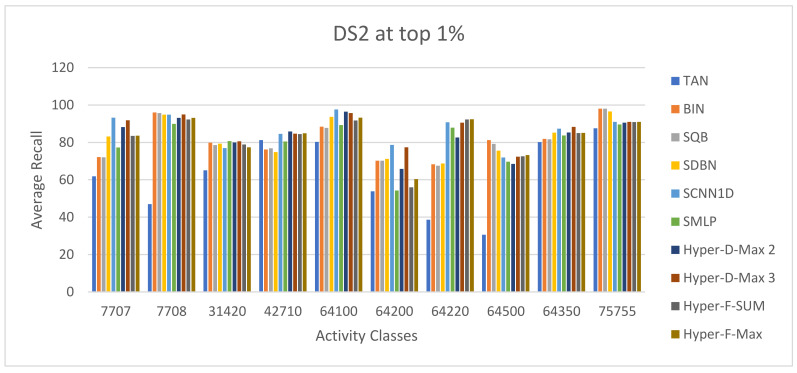
The comparison among methods for the average recall percentage at the top 1% in MDDR-DS2 (homogeneous).

**Figure 11:**
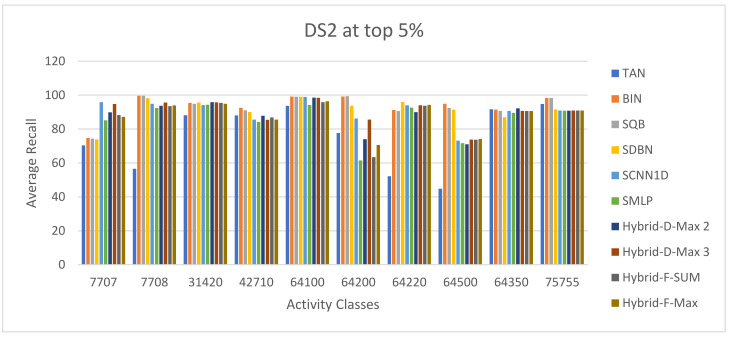
The comparison among methods for the average recall percentage at the top 5% in MDDR-DS2 (homogeneous).

**Figure 12:**
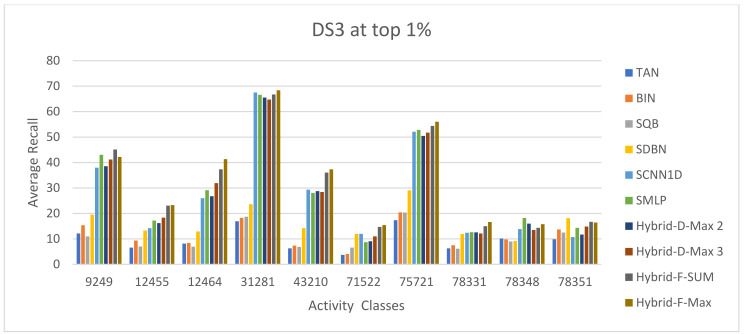
The comparison among methods for the average recall percentage at the top 1% in MDDR-DS3 (structurally heterogeneous).

**Figure 13:**
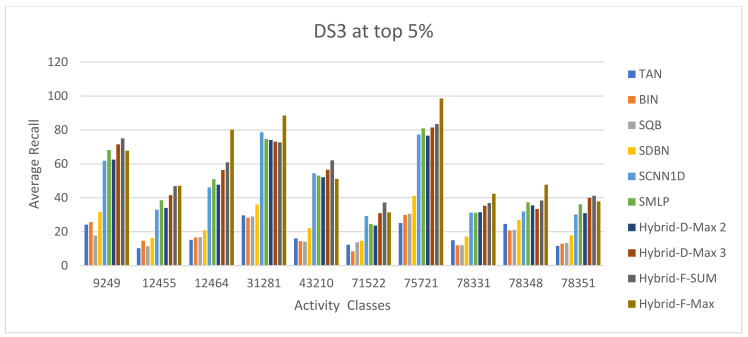
The comparison among methods for the average recall percentage at the top 5% in MDDR-DS3 (structurally heterogeneous).

**Figure 14:**
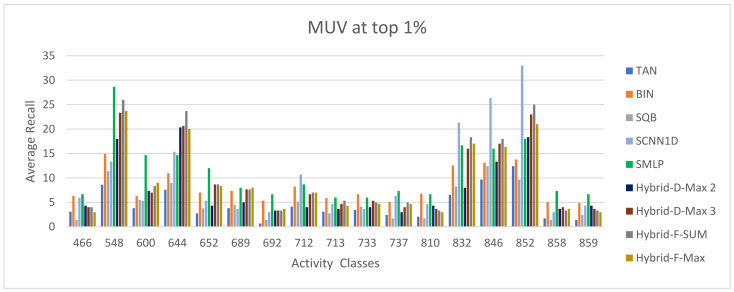
The comparison among methods for the average recall percentage at the top 1% in MUV.

**Figure 15:**
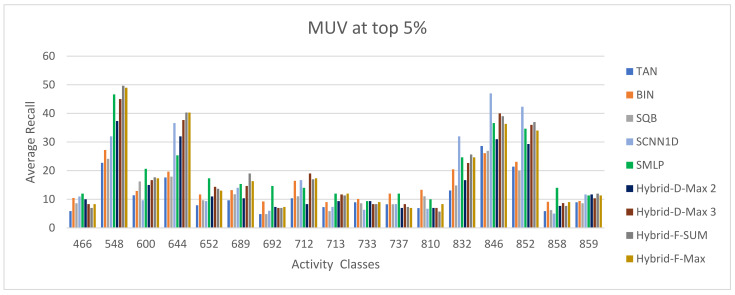
The comparison among methods for the average recall percentage at the top 5% in MUV.

The ECFC-4 descriptor’s experimental outcomes on the MDDRDS1, MDDR-DS2, MDDR-DS3, and MUV data sets are provided in Table 5, Table 6, Table 7, Table 8, Table 9, Table 10, Table 11 and Table 12, respectively, using cut-offs 1 and 5%. In addition, the results of the proposed Hybrid Siamese Similarity Models are recorded in these tables compared to the benchmark Tanimoto Similarity Coefficient (TAN) and previous studies, which are Bayesian inference (BIN), quantum similarity search (SQB), Stack of deep belief networks (SDBN) in MDDR datasets only, and two of our proposal methods of Siamese architecture in previous studies, which are the SMLP similarity model and SCNN1D similarity model. The hybrid Siamese similarity model with decision fusion using two similarity measures is here called the Hybrid-D-Max2. The hybrid Siamese similarity model with decision fusion using three similarity measures is here called the Hybrid-D-Max3. The hybrid Siamese similarity model with feature fusion summation is here called the Hybrid-F-Sum. The hybrid Siamese similarity model with feature fusion max is here called the Hybrid-F-Max. Each row in the tables lists recall values for the top 1% and 5% of the activity class, and in each row, the best recall rate is shaded. In the tables, the mean row relates to the average of all activity classes, and the row of shaded cells is the total number of shaded cells with the top values for each technique over the full range of activity classes. The first column of the table represents the activity classes of the dataset. This is followed by four columns that represent the previous studies: TAN, BIN, SQR, and SDBN, and this is followed by two columns that represent the two proposed Siamese in previous studies: SMLP, and SCNN1D. It is then followed by four columns representing the proposed hybrid models in this study. Figure 8, Figure 9, Figure 10, Figure 11, Figure 12, Figure 13, Figure 14 and Figure 15 show the contrast among methods for the average recall percentage of successful compound retrieval at the top of the 1% and 5% in MDDRDS1, MDDRDS2, MDDRDS3, and MUV, respectively.

The results presented in MDDR-DS1 (structurally homogeneous and heterogeneous) recall values for the 1 and 5% cut-offs recorded in Table 5 and Table 6 showed that the proposed hybrids of Siamese similarity models were obviously superior to the benchmark studies: TAN, BIN, SQB, SDBN, and previous two selected proposed methods: Siamese SMLP and SCNN1D. In addition, among other hybrid Siamese similarity models, the Hyper model (Hybrid-F-Max) gives the best retrieval recall results in Table 5, in view of the mean and the number of shaded cells, followed by the Hyper model (Hybrid-F-Sum), in view of the mean, followed by the Hyper Siamese model (Hybrid-D-Max2) and the Hyper Siamese model (Hybrid-D-Max3), in view of the mean, followed by the proposed methods in objective one SCNN1D and SMLP, and followed by the SDNB, BIN, SQB, and TAN in view of the mean. The improvement percentages of the hybrid-F-Max model in the mean recall values compared with previous studies and the two proposed methods in objective one are 15.25, 21.09, 55.2, 48.28, 50.35, and 44.45 compared to SCNN1D, SMLPearly, TAN, BIN, SQB, and SDNB, respectively. The improvement percentages of the hybrid-F-Sum model are 9.23,15.48, 52.03, 44.60, 46.82, 40.50 compared to SCNN1D, SMLP, TAN, BIN, SQB, and SDNB, respectively. The improvement percentages of the hybrid-D-Max3 model are 0.41, 7.26, 47.37, 39.22, 41.65, 34.72 compared to SCNN1D, SMLP, TAN, BIN, SQB, and SDNB, respectively. The improvement percentages of the hybrid-D-Max2 model are 0.89, 7.71, 47.62, 39.52, 41.94, and 35.03 compared to SCNN1D, SMLP, TAN, BIN, SQB, and SDNB respectively. Figure 8 compares methods for the average recall percentage of successful compound retrieval at the top 1% in MDDR-DS1. By comparison, the Hybrid-F-Max proposed method gives the best retrieval recall results in Table 6 in view of the mean, and the number of shaded cells, followed by the Hybrid-F-Sum, Hybrid-D-Max 2, Hybrid-D-Max 3, SCNN1D, and SMLP. Then, SDNB, BIN, SQB, and TAN are in view of the mean. The improvement percentages of the hybrid-F-Max model are 15.50, 18.26, 49.49, 45.24, 48.42, and 40.69 compared to SCNN1D, SMLP, TAN, BIN, SQB, and SDNB, respectively. The improvement percentages of the hybrid-F-Sum model are 11.03, 13.94, 46.82, 42.35, 45.70, and 37.55 compared to SCNN1D, SMLP, TAN, BIN, SQB, and SDNB, respectively. The improvement percentages of the hybrid-D-Max3 model are 0.49, 3.74, 40.52, 35.51, 39.26, and 30.15 compared to SCNN1D, SMLP, TAN, BIN, SQB, and SDNB, respectively. The improvement percentages of the hybrid-D-Max2 model are 0.68, 3.92, 40.63, 35.64, 39.38, and 30.28 compared to SCNN1D, SMLP, TAN, BIN, SQB, and SDNB, respectively. Finally, Figure 9 compares methods for the average recall percentage of successful compound retrieval at the top 5% in MDDR-DS1.

Furthermore, the MDDR-DS2 (structurally homogeneous) recall values recorded for the top 1% in Table 7 show that some of the proposed Siamese similarity models’ proposed hybrids are superior to the benchmark TAN method and previous studies. The Hyper Siamese with the decision fusion max model with three similarity measures Hybrid-D-Max 3 gives the best retrieval recall results in Table 7 in view of the mean, followed by SCNN1D in view of the mean and the number of shaded cells, and followed by Hybrid-D-Max 2, Hybrid-F-Max, Hybrid-F-Sum, SDBN, BIN, SQB, SMLP, and TAN. The improvement percentages of the hybrid-D-Max3 model are 0.05, 7.42, 27.79, 6.34, 6.89, and 5.12 compared to SCNN1D, SMLP, TAN, BIN, SQB, and SDNB, respectively. The improvement percentages of the hybrid-D-Max2 model are 4.01, 25.13, 2.89, 3.46, and 1.62 compared to SMLP, TAN, BIN, SQB, and SDNB, respectively. The improvement percentages of the hybrid-F-Max model are 3.74, 24.92, 2.62, 3.18, and 1.34 compared to SMLP, TAN, BIN, SQB, and SDNB, respectively. The improvement percentages of the hybrid-F-Sum model are 2.97, 24.32, 1.84, 2.41, and 0.56 compared to SMLP, TAN, BIN, SQB, and SDNB, respectively. Figure 10 shows the comparison among methods for the average recall percentage of successful compound retrieval at the top 1% in MDDR-DS2. However, the MDDR-DS2 recall values recorded for 5% cut-offs in Table 8 show that the BIN method gave the best retrieval recall results in view of the mean and the number of shaded cells. The second best is SQB, followed by SDBN, Hybrid-D-Max 3, SCNN1D, Hybrid-D-Max 2, Hybrid-F-Max, Hybrid-F-Sum, Hybrid-D-Max 2, and finally, TAN in view of the mean values. The improvement percentages of the hybrid-D-Max model are 0.07, 5.36, and 16.24 compared to SCNN1D, SMLP, and TAN, respectively. The improvement percentages of the hybrid-D-Max2 model are 3.09 and 4.22 compared to SMLP and TAN. The improvement percentages of the hybrid-F-Max model are 2.51 and 13.71 compared to SMLP and TAN. The improvement percentages of the hybrid-F-Max model are 1.83 and 13.11 compared to SMLP and TAN. Figure 11 shows the comparison among methods for the average recall percentage of successful compound retrieval at the top 5% in MDDR-DS2.

Moreover, the MDDR-DS3 (structurally heterogeneous) recall values for the 1 and 5% cut-offs recorded in Table 9 and Table 10 demonstrated that the proposed hybrids Siamese similarity models were superior to the benchmark TAN method and other studies: TAN, BIN, SQB, SDBN, and previous two selected proposed methods: Siamese MLP and CNN1D. In addition, among other hybrid Siamese similarity models, the Hyper Siamese with Feature fusion Max model (Hybrid-F-Max) gives the best retrieval recall results in Table 9 in view of the mean and the number of shaded cells, followed by the Hyper Siamese with Feature fusion Sum model (Hybrid-F-Sum) in view of the mean, followed by SMLP, the Hybrid-D-Max3, SCNN1D, and the Hybrid-D-Max2, and followed by the SDNB, BIN, SQB, and TAN in view of the mean. The improvement percentages of the hybrid-F-Max model are 17.03, 12.64, 70.72, 65.68, 68.44, and 50.80 compared to SCNN1D, SMLP, TAN, BIN, SQB, and SDNB, respectively. The improvement percentages of the hybrid-F-Sum model are 14.62, 10.10, 69.87, 64.67, 67.52, and 49.37 compared to SCNN1D, SMLP, TAN, BIN, SQB, and SDNB, respectively. The improvement percentages of the hybrid-D-Max3 model are 4.06, 66.15, 60.31, 63.51, and 43.11 compared to SCNN1D, TAN, BIN, SQB, and SDNB, respectively. The improvement percentages of the hybrid-D-Max2 model are 64.66, 58.57, 61.91, and 40.61 compared to TAN, BIN, SQB, and SDNB, respectively. Figure 12 compares methods for the average recall percentage of successful compound retrieval at the top 1% in MDDR-DS3. By comparison, the Hybrid-F-Max proposed method gives the best retrieval recall results in Table 10 in view of the mean and the number of shaded cells, followed by the Hybrid-F-Sum in view of the mean, Hybrid-D-Max 3, SMLP, SCNN1D, and Hybrid-D-Max 2. Then, SDNB, TAN, BIN, and SQB are in view of the mean. The improvement percentages of the hybrid-F-Max model are 20.08, 16.35, 68.9, 69.00, 69.63, and 58.72 compared to SCNN1D, SMLP, TAN, BIN, SQB, and SDNB, respectively. The improvement percentages of the hybrid-F-Sum model are 14.62, 10.64, 66.78, 66.88, 67.56, and 55.91 compared to SCNN1D, SMLP, TAN, BIN, SQB, and SDNB, respectively. The improvement percentages of the hybrid-D-Max3 model are 8.94, 4.69, 64.57, 64.67, 65.40, and 52.97 compared to SCNN1D, SMLP, TAN, BIN, SQB, and SDNB, respectively. The improvement percentages of the hybrid-D-Max2 model are 60.67, 60.78, 61.59, and 47.79 compared to TAN, BIN, SQB, and SDNB, respectively. Finally, Figure 13 compares methods for the average recall percentage of successful compound retrieval at the top 5% in MDDR-DS3.

Furthermore, the MUV recall values for the 1% cut-offs recorded in Table 11 demonstrated that some proposed hybrid Siamese similarity models were superior to the benchmark TAN method and other studies: TAN, BIN, SQB, and previous selected proposed method SCNN1D, except the SMLP method. In addition, among other hybrid Siamese similarity models, the Hyper Siamese with Feature fusion Sum model (Hybrid-F-Sum) gives the best retrieval recall results in Table 11 in view of the mean, followed by the SCNN1D, Hybrid-D-Max3, Hybrid-F-Max, BIN, Hybrid-D-Max2, SQB, and TAN. The improvement percentages of the hybrid-F-SUM model are 3.05, 50.64, 19.97, and 55.95 compared to SCNN1D, SQB, BIN, and TAN, respectively. The improvement percentages of the hybrid-F-Max model are 46.02, 12.48, and 51.83 compared to SQB, BIN, and TAN, respectively. The improvement percentages of the hybrid-D-Max3 model are 46.79, 13.74, and 52.52 compared to SQB, BIN, and TAN, respectively. The improvement percentages of the hybrid-D-Max2 model are 33.08 and 40.29 compared to SQB and TAN. Figure 14 compares the methods for the average recall percentage of successful compound retrieval at the top 1% MUV. By comparison, the Hybrid-F-Sum proposed method gives the best recall results in Table 12 in view of the mean, followed by the Hybrid-F-Max in view of the mean, Hybrid-D-Max 3, SCNN1D, and Hybrid-D-Max 2. Then, BIN, SQB, and TAN are in view of the mean. The improvement percentages of the hybrid-F-SUM model are 7.17, 34.07, 22.10, and 38.63 compared to SCNN1D, SQB, BIN, and TAN, respectively. The improvement percentages of the hybrid-F-Max model are 5.82, 33.11, 20.97, and 37.73 compared to SCNN1D, SQB, BIN, and TAN, respectively. The improvement percentages of the hybrid-D-Max3 model are 4.33, 32.05, 19.72, and 36.75 compared to SCNN1D, SQB, BIN, and TAN, respectively. The improvement percentages of the hybrid-D-Max2 model are 17.61, 2.65, and 23.30 compared to SQB, BIN, and TAN, respectively. Figure 15 compares the methods for the average recall percentage of the successful compound retrieval at the top 1% in MUV.

The second method that can be used to evaluate the proposed methods is the significance test. The Kendall W is the significance test that will be used in this study. Moreover, Table 13, Table 14, Table 15 and Table 16 show the ranking of the hybrid Siamese similarity models (Hybrid-F-Max, Hybrid-F-Sum, Hybrid-D-Max3, Hybrid-D-Max 2) based on previous studies TAN, BIN, SQB, Siamese MLP, and CNN1D, using Kendall W test results for MDDR-DS1, MDDR-DS2, MDDR-DS3, and MUV at the top 1% and top 5%.

For all of the data sets used, the Kendall W test of the top 1% shows that the significance test (P) values are less than 0.05; this means that the hybrid-enhanced Siamese similarity models are significant in all cases with the top 1%. Therefore, the general ranking of all methods indicates that the Hyper Siamese with Feature fusion Max model (Hybrid-F-Max) and the Hyper Siamese with Feature fusion Sum model (Hybrid-F-Sum) are superior to other methods and have the top rank in MDDR-DS1 (homogeneous and heterogeneous) and MDDR-DS3 (structurally heterogeneous). In MDDR-DS2 (structurally homogeneous), the hyper Siamese with the decision fusion max model with three similarities (Hybrid-D-Max3) has the top rank among other methods. In the MUV dataset, the Hyper Siamese with the Feature fusion Sum model (Hybrid-F-Sum) has the top rank among other methods except the SMLP method.

**Figure 16:**
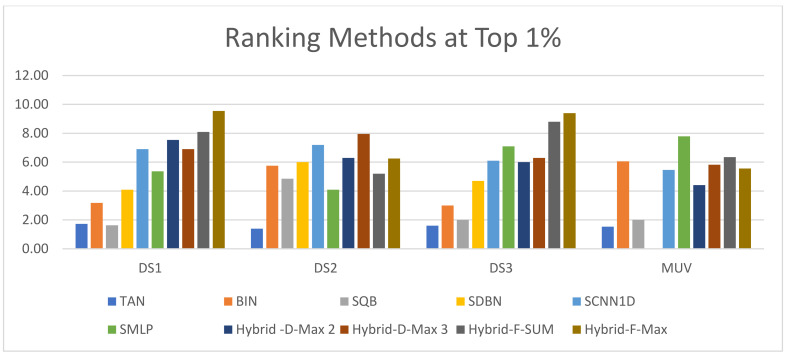
The ranking methods at the top 1%.

**Figure 17:**
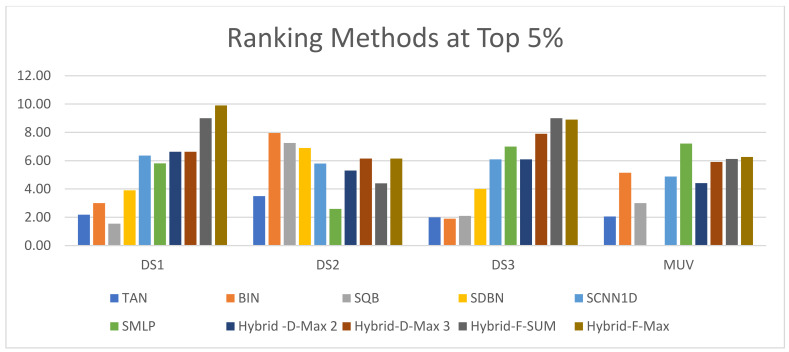
The ranking methods at the top 5%.

It is the same as with the results of the Kendall W test of the top 5%. The results indicate that significance test (P) values are less than 0.05. This means that the hybrid Siamese similarity models are significant in all cases with the top 5%. As a result, the general ranking of all methods indicates that the Hyper Siamese with Feature fusion Max model (Hybrid-F-Max) and the Hyper Siamese with Feature fusion Sum model (Hybrid-F-Sum) are superior to other methods and have the top rank in the MDDR-DS1(homogeneous and heterogeneous) and MDDR-DS3 (structurally heterogeneous). In DS2, BIN has the top rank in the top 5% and in the MUV dataset, the SMLP method has the top rank among other methods, and then the Hyper Siamese with Feature fusion Max model (Hybrid-F-Max). Figure 16 and Figure 17 show the ranking of the hybrid Siamese similarity models (Hybrid-D-Max2, Hybrid-D-Max3, Hybrid-F-Sum, Hybrid-F-Max) methods based on TAN, BIN, SQB, SDBN, Siamese MLP, and CNN1D using Kendall W test results for MDDR-DS1, MDDR-DS2, MDDR-DS3, and MUV in the top 1% and 5%, respectively.

Lastly, according to the experiment results, the success of the proposed methods comes from: (1) The Siamese network, which is used for more complicated data samples, especially with heterogeneous data samples, and it is possible to employ deep learning methods with Siamese architecture, which deals efficiently with the vast volume of information stored in databases. (2) Enhancing the Siamese architecture with several similarity measures because each similarity measure focused on different properties, so, when used together, they lead to an improvement in the recall metric. (3) Incorporate the two selected models in one hybrid model because each method provides good results in some classes, so combining them in one hybrid model improved the retrieval recall. The two designs of hybrid models, which used feature data fusion (Hybrid-F-Max and the Hybrid-F-Sum), gave good results compared with the other two designs of hybrid models, which used decision data fusion (Hybrid-D-Max3 and Hybrid-D-Max2) because the first two designs worked on the features, which are enhanced by using the sum and max operation, and then led to improvements in the recall metric. In comparison, the other two designs of hybrid models worked only on selecting the max results between the methods (SMLP, SCNN1D) in their hybrid designs.

Besides that, the proposed methods have good results in MDDR-DS3, MDDR-DS1, and MUV because they contain heterogeneous molecule classes. In MDDR-DS2, the proposed methods did not achieve a higher score than other traditional methods (TAN, BIN, SQB, SDBN) because the dataset has only structurally homogeneous molecules classes. However, some proposed methods have achieved better results at the top 1% only compared with traditional methods.

## 5. Conclusions

Many techniques for capturing the biological similarity between a test compound and a known target ligand in LBVS have been established. The similarity search is one of the primary tasks in VS that estimates a molecule’s similarity. It is predicated on the idea that molecules with similar structures may also have similar activities. In spite of the good performance of the methods, especially when dealing with molecules that have homogeneous active structural elements, they are not good enough when dealing with structurally heterogeneous molecules. The previous works examined many deep learning methods in the enhanced Siamese similarity model. According to Kendall W’s significant test, the best two methods in MDDR-DS3 (structurally heterogeneous) are the SMLP similarity model and the SCNN1D similarity model. To further improve the retrieval effectiveness of the similarity model, we incorporate the best two models in one hybrid model. The reason is that each method gives good results in some classes, so combining them in one hybrid model may improve the retrieval recall. Many designs of the hybrid models have been tested in this study. The overall results of all methods indicate that the Hybrid-F-Max method and the Hybrid-F-Sum method are superior to previous studies in DS1 and DS3 and have the top ranks among other methods at the top 1 and 5%, while the Hybrid-D-Max3, Hybrid-D-Max2, and Hybrid-F-Max are superior to previous studies in DS2 at the top 1%. In MUV, SMLP has the top rank, then Hybrid-F-Sum in the top 1%, and Hybrid-F-Max, Hybrid-F-Max, and Hybrid-D-Max3. The future work of this study is to reduce the size of the hybrid-enhanced Siamese similarity model by pruning the less significant weights.

## Figures and Tables

**Table 1:** The MDDR-DS1 (structurally homogeneous and heterogeneous) activity classes.

Activity Class	Activity Index	Active Molecules	Pairwise Similarity
Renin inhibitors	31420	1130	0.290
HIV protease inhibitors	71523	750	0.198
Thrombin inhibitors	37110	803	0.180
Angiotensin II AT1 antagonists	31432	943	0.229
Substance P antagonists	42731	1246	0.149
5HT3 antagonist	06233	752	0.140
5HT reuptake inhibitors	06245	359	0.122
D2 antagonists	07701	395	0.138
5HT1A agonists	06235	827	0.133
Protein kinase C inhibitors	78374	453	0.120
Cyclooxygenase inhibitors	78331	636	0.108

**Table 2:** The MDDR-DS2 (structurally homogeneous) activity classes.

Activity Class	Activity Index	Active Molecules	Pairwise Similarity
Adenosine (A1) agonists	07707	207	0.229
Adenosine (A2) agonists	07708	156	0.305
Renin inhibitors	31420	1130	0.290
CCK agonists	42710	111	0.361
Monocyclic lactams β	64100	1346	0.336
Cephalosporins	64200	113	0.322
Carbacephems	64220	1051	0.269
Carbapenems	64500	126	0.260
Tribactams	64350	388	0.305
Vitamin D analogous	75755	455	0.386

**Table 3:** The MDDR-DS3 (structurally heterogeneous) activity classes.

Activity Class	Activity Index	Active Molecules	Pairwise Similarity
Muscarinic (M1) agonists	09249	900	0.111
NMDA receptor antagonists	12455	1400	0.098
Nitric oxide synthase inhibitors	12464	505	0.102
Dopamine -hydroxylase inhibitors	31281	106	0.125
Aldose reductase inhibitors	43210	957	0.119
Reverse transcriptase inhibitors	71522	700	0.103
Aromatase inhibitors	75721	636	0.110
Cyclooxygenase inhibitors	78331	636	0.108
Phospholipase A2 inhibitors	78348	617	0.123
Lipoxygenase inhibitors	78351	2111	0.113

**Table 4:** MUV structure activity classes.

Activity Class	Activity Index	Pairwise Similarity
S1P1 rec. (agonists)	466	0.117
Rho-Kinase2 (inhibitors)	644	0.122
SF1 (inhibitors)	600	0.123
Eph rec. A4 (inhibitors)	689	0.113
HIV RT-Rnase (inhibitors)	652	0.099
HSP 90 (inhibitors) 30	712	0.106
SF1 (agonists)	692	0.114
ER-b-Coact. Bind. (inhibitors)	733	0.114
ER-a-Coact. Bind. (inhibitors)	713	0.113
FAK (inhibitors)	810	0.107
ER-a-Coact. Bind. (potentiators)	737	0.129
FXIa (inhibitors)	846	0.161
Cathepsin G (inhibitors)	832	0.151
D1 rec. (allosteric modulators)	858	0.111
FXIIa (inhibitors)	852	0.150
PKA (inhibitors)	548	0.128
M1 rec. (allosteric inhibitors)	859	0.126

**Table 5:** Top 1% retrieval recall for MDDR-DS1 (structurally homogeneous and heterogeneous) dataset for descriptor (ECFC 4).

DS1	Previous Studies				Previous in Our Work		Proposed Methods			
Retrieval Result 1%										
Activity Index										
	TAN	BIN	SQB	SDBN	SCNN1D	SMLP	Hybrid-D-Max 2	Hybrid-D-Max 3	Hybrid-F-SUM	Hybrid-F-Max
31420	69.69	74.08	73.73	74.21	84.58	84.19	84.22	83.88	86.94	88.28
71523	25.94	28.26	26.84	27.97	59.41	61.25	60.53	61.48	59.02	66.45
37110	9.63	26.05	24.73	26.03	52.88	46.56	52.94	50.28	46.42	58.13
31432	35.82	39.23	36.66	39.79	66.41	64.19	68.33	68.18	78.8	69.72
42731	17.77	21.68	21.17	23.06	38.88	42.69	42.19	42.81	34.02	55.45
6233	13.87	14.06	12.49	19.29	35.03	23.87	33.36	35.64	32.66	47.93
6245	6.51	6.31	6.03	6.27	10.68	6.79	10.9	10.85	19.02	16.25
7701	8.63	11.45	11.35	14.05	16.96	14.78	14.89	14.86	29.86	22.03
6235	9.71	10.84	10.15	12.87	15.31	12.82	16.32	14.29	26.97	23.58
78374	13.69	14.25	13.08	17.47	24.6	21.78	24.67	24.31	25.53	25.89
78331	7.17	6.03	5.92	9.93	8.58	5.94	8.69	8.44	16.12	14
**Mean**	**19.857**	**22.931**	**22.014**	**24.631**	**37.57**	**34.99**	**37.91**	**37.73**	**41.39**	**44.34**
**Shaded cells**	0	0	0	1	2	2	0	0	5	6

**Table 6:** Top 5% retrieval recall for MDDR-DS1 (structurally homogeneous and heterogeneous) dataset for descriptor (ECFC 4).

DS1	Previous Studies				Previous in Our Work		Proposed Methods			
Retrieval Result 5%										
Activity Index										
	TAN	BIN	SQB	SDBN	SCNN1D	SMLP	Hybrid-D-Max 2	Hybrid-D-Max 3	Hybrid-F-SUM	Hybrid-F-Max
31420	83.49	87.61	87.22	89.03	87.35	90.82	87.55	87.58	90.43	94.06
71523	48.92	52.72	48.7	65.17	79.61	79.48	79.51	80.65	83.44	86.44
37110	21.01	48.2	45.62	41.25	76	73.91	76.55	75.56	81.71	84.54
31432	74.29	77.57	70.44	79.87	91.83	93.87	93.81	92.26	95.23	95.02
42731	29.68	26.63	24.35	31.92	57.52	61.06	61.2	62.53	69.74	77.27
6233	27.68	23.49	20.04	29.31	62.76	53.57	62.67	64.21	75.23	80.2
6245	16.54	14.86	13.72	21.06	28.9	20.9	27.94	28.54	34.79	39.35
7701	24.09	27.79	26.73	28.43	42.25	38.33	38.43	39.82	46.68	49.65
6235	20.06	23.78	22.81	27.82	40.36	35.98	39.93	37.3	51.65	53.21
78374	20.51	20.2	19.56	19.09	48.27	48.4	49.44	47.78	55.27	57.82
78331	16.2	11.8	11.37	16.21	25.02	22.65	27.23	26.77	35.06	39.69
**Mean**	**34.77**	**37.7**	**35.51**	**40.83**	**58.17**	**56.27**	**58.57**	**58.45**	**65.38**	**68.84**
**Shaded cells**	0	0	0	0	0	0	0	0	1	10

**Table 7:** Top 1% retrieval recall for MDDR-DS2 (structurally homogeneous) dataset for descriptor (ECFC 4).

DS2	Previous Studies				Previous in Our Work		Proposed Methods			
Retrieval Result 1%										
Activity Index										
	TAN	BIN	SQB	SDBN	SCNN1D	SMLP	Hybrid-D-Max 2	Hybrid-D-Max 3	Hybrid-F-SUM	Hybrid-F-Max
7707	61.84	72.18	72.09	83.19	93.27	77.32	88.2	91.8	83.46	83.61
7708	47.03	96	95.68	94.82	94.84	89.94	93.16	94.9	92.32	93.1
31420	65.1	79.82	78.56	79.27	76.96	80.66	80.02	80.64	78.93	77.35
42710	81.27	76.27	76.82	74.81	84.55	80.55	85.82	84.73	84.45	84.91
64100	80.31	88.43	87.8	93.65	97.63	89.33	96.47	95.66	91.73	93.22
64200	53.84	70.18	70.18	71.16	78.65	54.26	65.87	77.35	55.94	60.39
64220	38.64	68.32	67.58	68.71	90.81	87.91	82.62	90.53	92.29	92.34
64500	30.56	81.2	79.2	75.62	71.92	69.68	68.56	72.4	72.56	73.2
64350	80.18	81.89	81.68	85.21	87.32	83.66	85.27	88.34	84.99	85.06
75755	87.56	98.06	98.02	96.52	90.95	89.65	90.53	90.99	90.9	90.99
**Mean**	**62.633**	**81.235**	**80.761**	**82.296**	**86.69**	**80.3**	**83.65**	**86.73**	**82.76**	**83.42**
**Shaded cells**	0	3	0	0	3	1	1	1	0	1

**Table 8:** Top 5% retrieval recall for MDDR-DS2 (structurally homogeneous) dataset for descriptor (ECFC 4).

DS2	Previous Studies				Previous in Our Work		Proposed Methods			
Retrieval Result 5%										
Activity Index										
	TAN	BIN	SQB	SDBN	SCNN1D	SMLP	Hyper-D-Max 2	Hybrid-D-Max 3	Hybrid-F-SUM	Hybrid-F-Max
7707	70.39	74.81	74.37	73.9	95.85	85.17	89.9	94.83	88.29	87.17
7708	56.58	99.61	99.61	98.22	94.9	92.45	93.74	95.61	93.48	93.94
31420	88.19	95.46	94.88	95.64	94.12	94.42	95.82	95.72	95.49	94.89
42710	88.09	92.55	91.09	90.12	85.64	84.18	87.82	85.45	86.91	85.64
64100	93.75	99.22	99.03	99.05	98.93	94.21	98.58	98.39	95.92	96.41
64200	77.68	99.2	99.38	93.76	86.19	61.48	74	85.61	63.48	70.58
64220	52.19	91.32	90.62	96.01	94.07	92.62	89.93	94.04	93.72	94.28
64500	44.8	94.96	92.48	91.51	73.2	71.68	71.04	73.84	73.76	74.16
64350	91.71	91.47	90.78	86.94	90.7	89.58	92.26	90.7	90.6	90.65
75755	94.82	98.35	98.37	91.6	90.99	90.86	90.84	90.99	90.95	90.99
**Mean**	**75.82**	**93.695**	**93.061**	**91.675**	**90.46**	**85.67**	**88.39**	**90.52**	**87.26**	**87.87**
**Shaded cells**	1	4	3	1	1	0	2	0	0	0

**Table 9:** Top 1% retrieval recall for MDDR-DS3 (structurally heterogeneous) dataset for descriptor (ECFC 4).

Ds3 Retrieval Result 1%	Previous Studies				Previous in Our Work		Proposed Methods			
Activity Index										
	TAN	BIN	SQB	SDBN	SCNN1D	SMLP	Hybrid-D-Max 2	Hybrid-D-Max 3	Hybrid-F-SUM	Hybrid-F-Max
9249	12.12	15.33	10.99	19.47	38.01	43.06	38.53334	41.16666	45.12	42.19
12455	6.57	9.37	7.03	13.29	14.21	17.22	16.24286	18.37856	23.09	23.26
12464	8.17	8.45	6.92	12.91	25.98	29.13	26.79208	31.94058	37.31	41.33
31281	16.95	18.29	18.67	23.62	67.52	66.57	65.52382	64.7619	66.76	68.38
43210	6.27	7.34	6.83	14.23	29.34	28.08	28.78536	28.37696	36.05	37.35
71522	3.75	4.08	6.57	11.92	12	8.71	9.08571	10.985706	14.7	15.43
75721	17.32	20.41	20.38	29.08	52.11	52.83	50.48818	51.73228	54.43	56.06
78331	6.31	7.51	6.16	11.93	12.41	12.65	12.56694	12.125994	14.96	16.65
78348	10.15	9.79	8.99	9.17	13.85	18.18	15.999974	13.512192	14.33	15.79
78351	9.84	13.68	12.5	18.13	10.71	14.34	11.715648	14.8673	16.68	16.42
**Mean**	**9.745**	**11.425**	**10.504**	**16.375**	**27.62**	**29.08**	**27.57**	**28.78**	**32.34**	**33.29**
**Shaded cells**	0	0	0	1	0	1	0	0	1	7

**Table 10:** Top 5% retrieval recall for MDDR-DS3 (structurally heterogeneous) dataset for descriptor (ECFC 4).

Ds3 Retrieval Result 5%	Previous Studies				Previous in Our Work		Proposed Methods			
Activity Index										
	TAN	BIN	SQB	SDBN	SCNN1D	SMLP	Hybrid-D-Max 2	Hybrid-D-Max 3	Hybrid-F-SUM	Hybrid-F-Max
9249	24.17	25.72	17.8	31.61	61.84	68.2	62.47778	71.52222	75.03	67.78
12455	10.29	14.65	11.42	16.29	32.97	38.59	34.05	41.58572	46.95	47.07
12464	15.22	16.55	16.79	20.9	46.12	51.01	47.70298	56.37622	60.95	80.2
31281	29.62	28.29	29.05	36.13	78.57	74.76	74.09524	73.14286	72.67	88.57
43210	16.07	14.41	14.12	22.09	54.47	53.08	52.09424	56.50262	62.08	51.15
71522	12.37	8.44	13.82	14.68	29.19	24.57	23.62858	30.95714	37.27	31.36
75721	25.21	30.02	30.61	41.07	77.31	80.99	76.6614	81.44882	83.46	98.66
78331	15.01	12.03	11.97	17.13	31.29	31.17	31.52754	35.32284	36.93	42.36
78348	24.67	20.76	21.14	26.93	31.89	37.33	35.51222	33.43086	38.41	47.8
78351	11.71	12.94	13.3	17.87	30.16	36.2	30.95734	40.02844	41.2	37.89
**Mean**	**18.43**	**18.38**	**18**	**24.47**	**47.38**	**49.59**	**46.87**	**52.03**	**55.5**	**59.28**
**Shaded cells**	0	0	0	0	1	0	0	0	4	6

**Table 11:** Top 1% retrieval recall for MUV dataset for descriptor (ECFC4).

MUV 1%	Previous Studies			Previous in Our Work		Proposed Methods			
Activity Index									
	TAN	BIN	SQB	SCNN1D	SMLP	Hybrid-D-Max 2	Hybrid-D-Max 3	Hybrid-F-SUM	Hybrid-F-Max
466	3.1	6.33	1.38	6	6.67	4.33	4.00	4.00	3.00
548	8.62	14.89	11.38	13.33	28.67	18.00	23.33	26.00	23.67
600	3.79	6.33	5.52	5.33	14.67	7.33	7.00	8.33	9.00
644	7.59	11	8.97	15.33	14.67	20.33	20.67	23.67	20.00
652	2.76	7	3.79	5.33	12.00	4.33	8.67	8.67	8.33
689	3.79	7.33	4.48	3.67	8.00	5.00	7.67	7.67	8.00
692	0.69	5.33	1.38	3	6.67	3.33	3.33	3.33	3.67
712	4.14	8.22	5.17	10.67	8.67	4.00	6.67	7.00	7.00
713	3.1	5.89	2.76	4.67	6.00	3.67	4.67	5.33	4.33
733	3.45	6.67	4.14	3.67	6.00	4.00	5.33	5.00	4.67
737	2.41	5.11	1.72	6.33	7.33	3.00	4.00	5.00	4.67
810	2.07	6.78	1.72	4.67	6.67	4.33	3.67	3.33	3.00
832	6.55	12.55	8.28	21.33	16.67	8.00	16.00	18.33	17.00
846	9.66	13.11	12.41	26.33	16.00	13.33	17.00	18.00	16.33
852	12.41	13.78	9.66	33	18.00	18.33	23.00	25.00	21.00
858	1.72	5.11	1.38	3	7.33	3.67	4.00	3.33	3.67
859	1.38	4.89	2.41	4.33	6.67	4.33	3.67	3.33	3.00
**Mean**	**4.54**	**8.25**	**5.09**	**10.00**	**11.22**	**7.61**	**9.57**	**10.31**	**9.43**
**Shaded cells**	**0**	**2**	**0**	**4**	**10**	**0**	**0**	**1**	**0**

**Table 12:** Top 5% retrieval recall for MUV dataset for descriptor (ECFC4).

MUV 5%	Previous Studies			Previous in Our Work		Proposed Methods			
Activity Index									
	TAN	BIN	SQB	SCNN1D	SMLP	Hybrid-D-Max 2	Hybrid-D-Max 3	Hybrid-F-SUM	Hybrid-F-Max
466	5.86	10.44	8.62	11	12.00	10.00	8.33	7.00	8.33
548	22.76	27.22	24.14	32	46.67	37.33	45.00	49.67	49.00
600	11.38	12.89	16.21	9.67	20.67	15.00	16.67	17.67	17.33
644	17.59	19.67	17.93	36.67	25.33	32.00	37.67	40.33	40.33
652	7.93	11.67	9.66	9.33	17.33	11.00	14.33	13.67	13.00
689	9.66	13.22	11.72	14	15.33	10.33	14.67	19.00	16.33
692	4.83	9.22	4.83	6	14.67	7.33	7.00	7.00	7.33
712	10.34	16.45	11.03	16.67	14.00	8.33	19.00	17.00	17.33
713	7.24	9	5.86	7.33	12.00	9.33	11.67	11.33	12.00
733	8.97	10.11	8.62	6.33	9.33	9.33	8.33	8.33	9.00
737	8.28	12	8.28	8.33	12.00	7.00	8.33	7.33	7.00
810	6.9	13.33	11.03	6.67	10.00	7.00	7.00	5.67	8.33
832	13.1	20.44	14.83	32	24.67	16.67	22.67	25.67	24.67
846	28.62	26.11	26.9	47	36.67	31.00	40.00	39.00	36.33
852	21.38	23.11	20	42.33	34.67	29.33	36.00	37.00	34.00
858	5.86	9.11	6.21	5	14.00	7.67	8.67	7.67	9.00
859	8.97	9.44	8.62	11.67	11.33	11.67	10.33	12.00	11.33
**Mean**	**11.75**	**14.91**	**12.62**	**17.76**	**19.45**	**15.31**	**18.57**	**19.14**	**18.86**
**Shaded cells**	**0**	**3**	**0**	**3**	**7**	**0**	**1**	**4**	**1**

**Table 13:** Ranking of hybrid Siamese similarity models based on (TAN, BIN, SQB, SDBN, SCNN1D, and SMLP) using Kendall W test results for DS1, at top 1% and 5%.

Dataset	Retrieval Percentage	W	P	Rank Methods	
DS1	1%	0.8214876	8.80 × 10^−14^	Hybrid-F-Max	9.55
				Hybrid-F-SUM	8.09
				Hybrid-D-Max 2	7.55
				Hybrid-D-Max 3	6.91
				SCNN1D	6.91
				SMLPearly	5.36
				SDBN	4.09
				BIN	3.18
				TAN	1.73
				SQB	1.64
	5%	0.8551465	1.91 × 10^−14^	Hybrid-F-Max	9.91
				Hybrid-F-SUM	9.00
				Hybrid-D-Max 3	6.64
				Hybrid-D-Max 2	6.64
				SCNN1D	6.36
				SMLPearly	5.82
				SDBN	3.91
				BIN	3.00
				TAN	2.18
				SQB	1.55

**Table 14:** Ranking of hybrid Siamese similarity models based on (TAN, BIN, SQB, SDBN, SCNN1D, and SMLP) using Kendall W test results for DS2, at top 1% and 5%.

Dataset	Retrieval Percentage	W	P	Rank Methods	
DS2	1%	0.3603155	1.68 × 10^−4^	Hybrid-D-Max 3	7.95
				SCNN1D	7.20
				Hybrid-D-Max 2	6.30
				Hybrid-F-Max	6.25
				SDBN	6.00
				BIN	5.75
				Hybrid-F-SUM	5.20
				SQB	4.85
				SMLPearly	4.10
				TAN	1.40
	5%	0.3082167	1.05 × 10^−3^	BIN	7.95
				SQB	7.25
				SDBN	6.90
				Hybrid-D-Max 3	6.15
				SCNN1D	5.80
				Hybrid-D-Max 2	5.30
				Hybrid-F-Max	5.15
				Hybrid-F-SUM	4.40
				TAN	3.50
				SMLPearly	2.60

**Table 15:** Ranking of hybrid Siamese similarity models based on (TAN, BIN, SQB, SDBN, SCNN1D, and SMLP) using Kendall W test results for DS3, at top 1% and 5%.

Dataset	Retrieval Percentage	W	P	Rank Methods	
DS3	1%	0.7789091	1.45 × 10^−11^	Hybrid-F-Max	9.40
				Hybrid-F-Sum	8.80
				SMLPearly	7.10
				Hybrid-D-Max 3	6.30
				SCNN1D	6.10
				Hybrid-D-Max 2	6.00
				SDBN	4.70
				BIN	3.00
				SQB	2.00
				TAN	1.60
	5%	0.8673939	3.91 × 10^−13^	Hybrid-F-Sum	9.00
				Hybrid-F-Max	8.90
				Hybrid-D-Max 3	7.90
				SMLPearly	7.00
				Hybrid-D-Max 2	6.10
				SCNN1D	6.10
				SDBN	4.00
				SQB	2.10
				TAN	2.00
				BIN	1.90

**Table 16:** Ranking of hybrid Siamese similarity models based on (TAN, BIN, SQB, SCNN1D, and SMLP) using Kendall W test results for MUV, at top 1% and 5%.

Dataset	Retrieval Percentage	W	P	Rank Methods	
MUV	1%	0.5593166	3.01 × 10^−13^	SMLP	7.79
				Hybrid-F-Sum	6.35
				BIN	6.06
				Hybrid-D-Max 3	5.82
				Hybrid-F-Max	5.56
				SCNN1D	5.47
				Hybrid-D-Max 2	4.41
				SQB	2.00
				TAN	1.53
	5%	0.362653	5.52 × 10^−8^	SMLP	7.21
				Hybrid-F-Max	6.26
				Hybrid-F-Sum	6.12
				Hybrid-D-Max 3	5.91
				BIN	5.15
				SCNN1D	4.88
				Hybrid-D-Max 2	4.41
				SQB	3.00
				TAN	2.06

## Data Availability

The MDL Drug Data Report (MDDR) dataset is owned by www.accelrys.com (accessed on 31 October 2021). A license is required to access the data. Maximum Unbiased Validation (MUV) Data Sets are freely available at http://www.pharmchem.tu-bs.de/lehre/baumann/MUV.html (accessed on 31 October 2021). Software License: Python 3.7 in environment anaconda/Spyder was used with the following libraries: TensorFlow, theano, keras, numpy, pandas and math. the license of statistics application (IBM spss) is licenseapp.utm.my.
